# Values, Attitudes Toward Interpersonal Violence, and Interpersonal Violent Behavior

**DOI:** 10.3389/fpsyg.2018.00604

**Published:** 2018-05-09

**Authors:** Daniel Seddig, Eldad Davidov

**Affiliations:** ^1^Institute of Sociology and Social Psychology, University of Cologne, Cologne, Germany; ^2^Department of Psychology, University of Zurich, Zürich, Switzerland; ^3^Department of Sociology, University of Zurich, Zürich, Switzerland

**Keywords:** violent behavior, basic human values, attitudes, emerging adulthood, structural equation modeling

## Abstract

The relevance of human values for the study of the motivational sources of interpersonal violent behavior was investigated in various fields of the social sciences. However, several past studies mixed up values with other dimensions like attitudes, norms, or beliefs, and only a few systematically assessed the effect of values on violent behavior relying on a value theory. Furthermore, in other studies, violence was often analyzed as a composite index of different forms of delinquent behavior rather than as violence *per se*. In the current study we address these gaps in the literature by building upon Schwartz’ theory of basic human values. We use it to explain attitudes toward interpersonal violence and interpersonal violent behavior. We analyze data of young people (*n* = 1,810) drawn from a German study in Duisburg, Germany, which assessed various types of self-reported violent behavior as well as values and attitudes toward violence. We test structural equation models in which we explain interpersonal violent behavior with basic human values, and where attitudes toward interpersonal violent behavior mediate this relation. Results show that self-transcendence and conservation values are associated negatively and power and stimulation values positively with interpersonal violent behavior. In addition, attitudes operate as a partial mediator for the former and as a full mediator for the latter in the relation between values and violent behavior. Despite a dominant association between attitudes and behavior, values themselves can significantly contribute to the explanation of violent behavior.

## Introduction

Interpersonal violent behavior is a social problem that policymakers and researchers from various disciplines alike have tried to understand and resolve for a long time. Sociological and social psychological studies have put great effort into explaining interpersonal violent behavior. Many of these studies agree that individual values can potentially explain the support and prevalence of violent conduct (for earlier studies see, e.g., Cohen, 1955; Matza and Sykes, 1961; Lerman, 1968; Merton, 1968; Hirschi, 1969; for newer ones, see, e.g., Knafo, 2003; Knafo et al., 2008; Bilsky and Hermann, 2016). However, although the commonality of these studies is the use of values as explanatory variables of violent behavior, some (particularly earlier studies) mixed up values with other explanatory variables such as opinions, norms, or beliefs or used the value concept without any systematic consideration of a value theory (Cohen, 1955; Matza and Sykes, 1961; Lerman, 1968; Merton, 1968; Hirschi, 1969). This neglect is unfortunate because values can prove to be important individual explanations for violent behavior. Furthermore, some of the studies did not explain violent behavior *per se*, but classified it under a general category of delinquent behavior. However, the motivational basis for interpersonal violent behavior may be fundamentally different from that of other types of delinquent behavior (such as theft or tax evasion).

In the current study, we address this gap by building upon Schwartz (1992, 1994) theory of basic human values, which is one of the most systematically developed and empirically tested value theories to date, and by using it to explain interpersonal violent behavior. Based on the definitions and implications of the theory, we present the underlying mechanisms for the relations between specific values, attitudes toward violence, and violent behavior. We test the expected relationships with population data from a sample of young people in Germany using structural equation models. Before turning to the empirical parts in Sections “Data and Measures” and “Results”, we present our theoretical considerations in Sections “Basic Human Values, Attitudes, and Behavior” and “Values and Interpersonal Violence”, respectively.

## Basic Human Values, Attitudes, and Behavior

**FIGURE 1:**
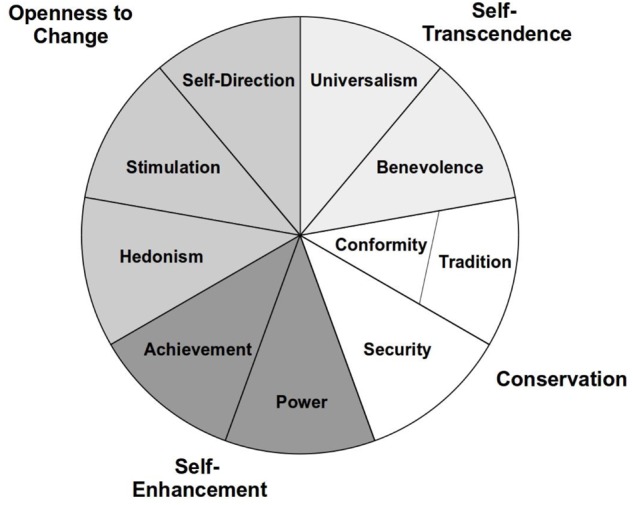
Circular structure of basic human values (based on Schwartz, 1992).

Based on the early work of Rokeach (1973), Schwartz (1992, 1994) developed his theory of basic human values.^1^ He defines values as desirable goals that are effective across situations, ordered relative to their importance, and direct an individual’s choice and evaluation of behavior, people, and events.^2^ Values emerge from organic biological needs, requisites of coordinated social interaction, and survival and welfare needs of groups. Schwartz identified 10 distinct values, each expressing a motivational aspect that is more or less important to the individual. *Universalism* refers to tolerance and protection of the needs of others, care for nature, and a sense for justice. *Benevolence* is concerned with loyalty as well as the preservation and enhancement of the welfare of those whom one is close to. *Tradition* emphasizes commitment to the customs and ideas from one’s culture and religion as well as personal restraint and modesty. *Conformity* describes the adjustment of behavior to social norms and the expectations of others as well as self-restraint. *Security* expresses the need for harmony, social stability, and family and personal safety. *Power* implies the goals of prestige, social status, and control over other people and resources. *Achievement* is concerned with personal success and demonstration of competence. *Hedonism* refers to the motivational need for pleasure, enjoyment, and the sensual satisfaction of short-term desires. *Stimulation* expresses the desire for arousal and excitement as well as for change and novelty in life. Finally, *self-direction* involves creativity, freedom, autonomy, and independent thinking and action. The theory also assumes that the 10 values are arranged in a circular structure (see **Figure 1**). Adjacent values are assumed to share a common motivational basis and to be highly related to each other. Values on opposite sides of the circle are of a different motivational type, and their relationship is assumed to be small or even negative.

Individuals are generally motivated to maintain consistency between their values and actions (Rokeach, 1973; Bardi and Schwartz, 2003). If a behavior expresses the content of a particular value (i.e., a “value-expressive behavior,” see, e.g., Bardi and Schwartz, 2003; Schwartz and Butenko, 2014), the relationship between that value and the behavior is likely to be positive. The relationship is assumed to be strong when values are able to distinguish between behaviors that are relevant for the attainment of goals from other behaviors. If a value has a high priority for an individual, it is more likely that this person will perform a behavior which realizes the motivation behind that value (Cieciuch et al., 2015). An extensive body of empirical research shows support for the relationship between values and behavior (Roccas and Sagiv, 2017).

**FIGURE 2:**
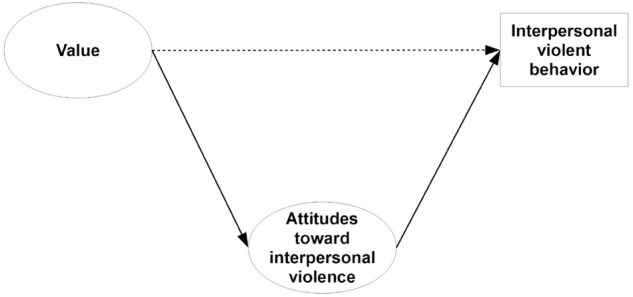
A general model for the relation between values, attitudes toward interpersonal violence, and interpersonal violent behavior.

However, attitudes may mediate the relationship between values and behavior. Attitudes are summary evaluations of specific (social psychological) objects, such as behavior, persons, institution, or events (Ajzen, 2001) and they serve as major determinants of intention and behavior in the theory of planned behavior (Ajzen, 1991). Values may influence not only behavior, but also attitudes, especially when they express the content of the value (“value-expressive attitudes”; see Maio and Olson, 2000; Maio, 2010).^3^ The underlying mechanism between values and attitudes relates to the attainment of motivational goals that are either promoted or suppressed by the attitude object (e.g., behavior; Sagiv and Schwartz, 1995; see also Davidov et al., 2008a). If the object helps to realize the motivational goals behind a value, the value–attitude link is expected to be positive. If the object blocks the motivational goals, the value–attitude link may be negative. However, the more attitudes correspond to a specific behavior, the more their influence on behavior is expected to exceed the influence of values (Homer and Kahle, 1988; Feather, 1995; Schwartz et al., 2010; Boer and Fischer, 2013). Therefore, we expect the relation between values and interpersonal violent behavior to be fully mediated by attitudes toward interpersonal violent behavior, and we test this expectation empirically. **Figure 2** illustrates the possible causal relation between values, attitudes, and behavior. It should be noted that both attitudes and behavior are not necessarily influenced by only one single value. Various values could exert an influence on attitudes and behavior simultaneously (Bardi and Schwartz, 2003).

Finally, a word of caution. We do not assess causality in this study in a strict sense. Indeed, consequences of behavioral decisions and attitudes may also have feedback effects on values (Vecchione et al., 2016). Thus, our findings may reflect evidence for associations rather than unidirectional causality. However, the proposed causal linkage between values, attitudes, and behavior lies on strong theoretical reasoning. We have solid reasons to believe that associations between values, attitudes, and behavior are at least indicative of some causal chain (Schwartz et al., 2017).^4^

## Values and Interpersonal Violence

Violent behavior can be considered a subcategory of aggressive behavior (Anderson and Bushman, 2002; Bushman and Huesmann, 2010). Aggression is a behavior that is performed with the intention to cause immediate harm to another person. The offender believes that the behavior will in fact cause harm and the potential victim is motivated to avoid the aggression. Although aggression is always overt and observable, it can appear in one of two forms (Archer and Coyne, 2005; Card et al., 2008): direct (e.g., slapping a person in the face, threating, mocking, name-calling) or indirect (e.g., gossiping, manipulation of the victim’s social status and relationships).^5^

Violent behavior is always direct and has extreme consequences (e.g., death or injury). Thus, not all forms of aggression are violent, but all forms of violence are aggressive. All forms of aggression and violence appear as a social behavior and involve an interaction between at least two persons. These criteria exclude a range of phenomena from interpersonal aggressive and violent behavior, such as violence directed toward inanimate objects without the intention to harm another person (e.g., hitting a wall), aggressive emotions (e.g., anger, hate), or cognitions (e.g., beliefs or attitudes toward violence). Hence, we refer to *interpersonal violence as overt acts that are intentionally carried out to cause physical harm to another person, who is motivated to avoid physical harm*.

Previous studies systematically demonstrate associations between various values and interpersonal violent behavior. Cole et al. (2007) found universalism, security, tradition, and conformity to be associated with lower rates of self-reported delinquent behaviors, and power and hedonism to be positively related with self-reported fighting among Bahamian adolescents. Knafo (2003) and Knafo et al. (2008) found universalism and conformity values to be negatively related and power to be positively related to self-report of violent or bullying behavior among Arab and Jewish adolescents in Israel. Menesini et al. (2013) found that self-reported physically harmful behavior was negatively related to the higher-order values conservation and self-transcendence and positively related to openness to change and self-enhancement among Italian pupils. Benish-Weisman and McDonald (2015) and Benish-Weisman (2015) used peer nominations in a sample of Israeli adolescents to assess aggressive behavior. Results showed that aggression is positively related with self-enhancement and openness to change values and negatively related with self-transcendence and conservation values. Finally, Bilsky and Hermann (2016) demonstrated that tradition and conformity are negatively and hedonism and stimulation positively related to self-reported delinquency of respondents in two German cities.

Based on these studies and our theoretical considerations, we expect to find an association between interpersonal violent behavior and most of the values. Universalism expresses concern for the welfare of others, and violence harms the welfare of others. Therefore, universalism is expected to have a negative impact on interpersonal behavior. Benevolence expresses concern for the welfare of those with whom one is close. Since its motivational goal is associated with ingroup harmony, friendship, honesty, helpfulness, and care for close others, we also expect benevolence to hinder the use of violence. Therefore, benevolence is expected to have a negative impact.

Tradition and conformity values are expected to have a negative impact as well. In most social contexts, violent behavior stands in contrast to traditional customs and social norms. Thus, the acceptance of traditional customs, modesty, and (even more) self-restraint and obedience to social norms are assumed to inhibit the evaluation of violence as an appropriate means of action. Similarly, security is also expected to have a negative impact on interpersonal behavior. Violent behavior poses a threat to the social order and to one’s personal and family security. Therefore, it is undesirable for individuals striving for order and security, and they are expected to refrain from violence or from supporting it.^6^

While universalism, benevolence, tradition/conformity, and security are expected to have a negative effect on interpersonal behavior, stimulation and power are expected to have a positive effect. Stimulation expresses a pursuit of excitement and risk-taking. Carrying out violent behavior can be both exciting and risky. Furthermore, violence may be regarded as a means to exercise control and power over people and resources and to gain respect. Thus, interpersonal violent behavior can prove itself to be beneficial for selfish purposes and as a useful means to gain power.

Achievement, which is located next to the power value on the value circle, is not expected to have any effect on interpersonal violence. On the one hand, ambition and success, which are important elements of achievement, may be enhanced by using violence. On the other hand, achievement is guided by social norms and conventional social standards, and most individuals motivated by this value are likely to conform to such standards. Therefore, overall we do not expect any association between achievement and interpersonal violence. Hedonism is also not expected to have any effect on interpersonal violent behavior. On the one hand, hedonism is concerned with seeking fun and pleasure. There are studies which link hedonism to psychopathy (e.g., Abraham and Rahardjo, 2015; Kajonius et al., 2015), showing that some individuals can find pleasure in violent behavior. On the other hand, violence is not comfortable or gratifying and neither fun nor pleasing for many other people. Therefore, overall we do not expect any relationship between hedonism and interpersonal violence. Finally, self-direction is not expected to have any effect. We see no mechanism to associate the goals of independence and creativity, which stand behind self-direction, with violence.

In sum, both previous studies and our theoretical considerations suggest single values that stand out with either positive or negative relations to aggression and interpersonal violence. Single values that stand out with negative relationships to interpersonal violence are universalism, tradition, conformity, and security. The strongest positive relationships to aggression and interpersonal violence are expected for power and stimulation values.

With the exception of Bilsky and Hermann (2016), all the previous studies discussed above assessed samples of adolescents. Adolescence is a dynamic period of social, cognitive, and neurobiological development that deserves special attention in the study of aggression and violence (see Benish-Weisman et al., 2017). The transition into adulthood (the so-called emerging adulthood, see Arnett, 2000, 2007) is a period of changes. Although violent behavior generally decreases with age (Farrington, 1986), it will not completely disappear in emerging adulthood. Therefore, our study contributes to the understanding of the behavior- and attitude-guiding functions of values in the period between adolescence and adulthood. Next, we will describe our dataset and test our theoretical expectations.

## Data and Measures

### Data

Data for the current analysis are taken from the “Crime in the modern city (Crimoc)” study (for details, see Boers et al., 2010; Seddig, 2014, 2016).^7^ The main objective of the study was to investigate the level and development of adolescent criminal behavior over time and into adulthood. The study began in 2002 in the city of Duisburg, Germany, targeting all seventh-grade pupils in the town.^8^ Forty out of 57 schools with a total of 3,910 pupils (consisting of 70% of the population of seventh-grade pupils) agreed to participate in the study (51% male; mean age 13.0 years). Response rates throughout the study ranged from 84 to 92 percent. At first, data collection took place annually by administering paper-and-pencil questionnaires during school time. In later waves, the mode of data collection was gradually changed to a postal questionnaire, because it was increasingly difficult to reach participants in schools as they grew older. Since the entry of participants into adulthood in 2009, data collection has been carried out biennially.

The current analysis is based on panel data from two waves (2011 and 2013) covering respondents at the ages of 22 and 24. The 2011 wave was the only wave in the study that included information to assess the basic human values of respondents. The sample of the Crimoc study did not focus on groups of individuals who are particularly engaged in delinquency and violence.^9^ Thus, the frequencies of self-reported delinquent and violent acts are considerably low, especially during the transition from adolescence to adulthood when processes of desistance from delinquency are effective (Bushway et al., 2003). To be able to analyze a sufficient number of violent acts, we combined self-reports of violent behavior from the years 2011 and 2013 into a single dataset. Since not all variables were measured at all times, the data unfortunately did not allow testing for cross-lagged effects over time for values, attitudes, and behavior.

The current sample consisted of *N* = 1,810 participants. The reduced number of respondents (compared to the number of respondents in the first wave in 2002) resulted from panel dropouts (Reinecke, 2013) and the anonymous code-based procedure to link the panel waves.^10^ We considered only those respondents who participated in both the 2011 and 2013 waves. Compared to the initial cohort sample in 2002, our sample had a lower percentage of males (38%), which may have led to an underestimation of the effects explaining violent behavior (see also Seddig, 2016).

Although our data is based on a panel study, we deliberately do not address processes of behavioral change for two reasons. First, data on values are available only for a single wave. Thus, the data does not allow examining how change in values may be related to change in violent behavior. Second, this question goes beyond the scope of the present study that looks at associations between value priorities, attitudes toward interpersonal violent behavior, and interpersonal violent behavior *per se*.

### Measures

**Table 1** displays all the items and their question wording (with mean values and standard deviations in parentheses).

**Table 1:** Items measuring values, attitudes, and behavior, and means (standard deviations in parentheses).

Dimension	Item	Meaning	Response categories	*M (SD)*
Universalism	(1)	Important that every person should be treated equally/have equal opportunities	1 - does not apply at all 5 - fully applies	4.07 (0.95)
	(2)	Important to listen to people who are different/understand them		3.86 (0.86)
	(3)	People should care for nature/environment is important to me		3.55 (0.99)
Benevolence	(4)	Important to help people around me/care for their well-being		3.91 (0.86)
	(5)	Important to be loyal to my friends/devoted to people that are close to me		4.22 (0.78)
Tradition	(6)	Important to be humble and modest/not to draw attention		3.14 (0.95)
	(7)	Tradition is important for me/follow customs of my religion, culture, and family		2.99 (1.17)
Conformity	(8)	People should do what they are told/follow rules at all times		3.23 (0.95)
	(9)	Important to behave properly/avoid doing anything people would say is wrong		3.25 (0.95)
Security	(10)	Important to live in secure surroundings/avoid anything that endangers my safety		3.61 (0.96)
	(11)	Important that government ensures safety/strong state so it can defend its citizens		3.89 (0.91)
Power	(12)	Important to be rich/to have money and expensive things		2.86 (0.95)
	(13)	Important to be respected/I want people to do what I say		2.63 (0.96)
Achievement	(14)	Important to show my abilities/receive admiration		3.44 (0.91)
	(15)	Important to be successful/recognition of my achievements		3.78 (0.89)
Hedonism	(16)	Important to have a good time/spoil myself		3.97 (0.83)
	(17)	I seek every opportunity to have fun/important to do things that give me pleasure		3.47 (0.89)
Stimulation	(18)	I like surprises and look for new things/important to do different things in life		3.62 (0.89)
	(19)	I look for adventures/an exciting life		2.80 (0.97)
Self-direction	(20)	Important to think up new ideas, be creative/do things in my own original way		3.60 (0.93)
	(21)	Important to make my own decisions/to be free and independent from others		4.07 (0.82)
Attitudes toward violence	(22)	I think that attacking another person and hitting her/him in the face is...	1 - very harmful 5 - totally harmless (recoded)	1.54 (0.78)
	(23)	I think that provoking and intimidating another person is...		2.06 (1.00)
	(25)	I think that robbing another person is...		1.23 (0.55)
Violent behavior	(26)	During the past 12 months: how often did you hit another person (with or without a weapon)/ snatch a bag from another person/ rob another person?	1 - reported at least one violent offense, 0 - reported no violent offense	0.04 (0.59)

#### Human Values

The questionnaire included 21 items similar to the Portrait Values Questionnaire (PVQ) items in the European Social Survey (ESS) to measure 10 values. They comprised two items to measure each value with the exception of universalism which was measured by three items (Schwartz et al., 2001; Davidov et al., 2008b). However, in contrast to the ESS questions, the items in the current study were not formulated as portraits, but rather as statements which participants rated on a scale from 1 (*does not apply at all*) to 5 (*fully applies*). For example, the two items for the value benevolence were formulated in the following way: “It is important to me to help people around me. I want to care for their well-being” and “It is important to me to be loyal to my friends. I want to devote myself for people close to me.” Thus, these measurements and operationalizations clearly tap into values rather than personality traits (see Schwartz et al., 2001).^11^

#### Attitudes Toward Violent Behavior

Participants expressed their evaluation of three types of violent behavior, that is, assault, intimidation, and robbery, by rating the behaviors’ harmfulness on a scale from 1 (*totally harmless*) to 5 (*very harmful*). We consider this an appropriate measurement of attitudes toward violent behavior because the evaluative responses as either harmless or harmful also carry a similarity to reactions such as favor or disfavor, approval or disapproval, or positivity or negativity.^12^ The items were recoded both in the analysis and in **Table 1** so that high values indicated a positive attitude toward violence. The distributions of responses were highly skewed. This is reflected by the proportions by which participants rated assault, intimidation, and robbery as harmless or totally harmless, which were 3.09%, 10.05%, and 0.94%, respectively.

#### Violent Behavior

Data on violent behavior consisted of self-reports about the frequency of robbery as well as two types of assault (with or without a weapon) conducted in the past year. All three behaviors are interpersonal in nature, implying that they are aimed against other people who may be known or unknown to the person carrying out the behavior. These types of behavior were conceptually closely related to the three measures of attitudes toward violent behavior. These behaviors implied the use of or at least the threat to use interpersonal physical violence. We created a single dichotomous behavioral variable with participants who reported (at least) one violent offense in one of the waves coded as 1 and otherwise with 0.^13^

The use of self-reports may be problematic for two reasons. Respondents may hide their true attitudes and behavior because the behavior is socially undesirable (Meyerl, 2013). Thus, self-reports may be downward biased and relations to other variables may be consequently underestimated. In addition, an assessment of values, attitudes, and behavior with the same measurement instrument may introduce a shared method bias (Kristof, 1996; Pozzebon and Ashton, 2009). However, contemporary studies on crime and deviance often reveal that self-report measures are nevertheless sufficiently valid and reliable for many research purposes (see, e.g., Thornberry and Krohn, 2000, 2003). Furthermore, the questionnaires were administered with the assurance of anonymity (no personal information was collected) which may have reduced social desirability to a minimum. Most participants were familiar with the panel study for many years, and it is reasonable to assume that they developed a degree of trust in the procedures, which minimized (but did not exclude) response bias. Finally, the distribution and developmental pattern of delinquent behavior in the present study resembled that found in other international studies which included similar self-reported offenses (Seddig and Reinecke, 2017).

A common issue in violence research is that data is not normally distributed, and that violent acts are rare events, especially in samples that do not focus on groups of individuals with a higher risk of delinquent involvement (e.g., males, convicts, individuals from urban areas with high crime rates). Furthermore, the violent events even decrease with age according to the age–crime curve (see Farrington, 1986). In the current data, 33 participants (1.8%) reported at least one violent offense at the age of 22 (wave 11). At the age of 24 (wave 13), 25 participants (1.4%) reported at least one violent offense. We estimated maximum-likelihood probit-coefficients (Muthén et al., 2016) to explain the variability in this variable in all subsequent structural equation models. With two predictors (specific values and attitudes) and nearly 50 events (i.e., about 25 events per predictor) non-linear estimates are considered trustworthy (Peduzzi et al., 1996; Vittinghoff and McCulloch, 2007).^14^

## Results

### Descriptive Correlations Between Values and Attitudes Toward Violent Behavior

We used confirmatory factor analysis (CFA) to estimate factor loadings, reliabilities, and correlations between the latent variables representing the values, the attitudes toward violent behavior, and violent behavior. We were not able to separate the tradition and conformity values due to very high correlations (standardized correlation greater than 1.0). We decided to unify the two values into a single value tradition/conformity and continue the analysis with nine values. The final CFA model fits the data well (χ^2^= 826.086; *df* = 212; RMSEA = 0.040; CFI = 0.922).^15^ The standardized factor loadings of all items and reliability coefficients are presented in **Table 2**. All factor loadings of the value items were greater than 0.40 with the exception of the tradition items “humble” (0.34) and “tradition” (0.39). The three measures of attitudes toward violence loaded strongly on the latent variable with standardized factor loadings of 0.79, 0.65, and 0.67, respectively. The omega coefficients were rather low for some of the value dimensions (e.g., self-direction) indicating that these measurements are only moderately reliable. However, the reliability estimates should be interpreted with caution because, with the exception of universalism and tradition/conformity, only two items were used to measure each value, and some of them were skewed (Raykov and Marcoulides, 2016). As displayed in **Table 3**, neighboring values correlated positively and opposing values correlated weakly, not at all, or negatively. The fact that opposing values were not always negatively correlated may be attributed to the fact that all values are desired (hence, hardly any negative correlations), but some are more desired than others are for different individuals (see, e.g., Davidov, 2008, 2010; Davidov et al., 2008b). Second, most values were significantly related to attitudes toward violence and violent behavior and in the expected direction. Specifically, universalism, benevolence, tradition/conformity, and security were negatively related and stimulation was positively related. Power, achievement, hedonism, and self-direction were either weakly or not significantly related with attitudes and behavior. Attitudes toward violent behavior and violent behavior were positively correlated.

**Table 2:** Standardized factor loadings and reliabilities.

Dimension	Item	Standardized factor loading	Reliability (omega)
Universalism	(1)	0.565 (0.021)	0.579 [0.540, 0.618]
	(2)	0.609 (0.019)	
	(3)	0.469 (0.022)	
Benevolence	(4)	0.880 (0.063)	0.514 [0.461, 0.568]
	(5)	0.534 (0.020)	
Tradition/Conformity	(6)	0.345 (0.027)	0.558 [0.523, 0.592]
	(7)	0.392 (0.025)	
	(8)	0.618 (0.023)	
	(9)	0.650 (0.022)	
Security	(10)	0.559 (0.022)	0.504 [0.455, 0.553]
	(11)	0.617 (0.023)	
Power	(12)	0.549 (0.024)	0.488 [0.438, 0.539]
	(13)	0.569 (0.027)	
Achievement	(14)	0.594 (0.019)	0.647 [0.609. 0.685]
	(15)	0.800 (0.020)	
Hedonism	(16)	0.707 (0.026)	0.576 [0.531, 0.620]
	(17)	0.412 (0.035)	
Stimulation	(18)	0.452 (0.025)	0.511 [0.438, 0.584]
	(19)	0.746 (0.024)	
Self-direction	(20)	0.403 (0.027)	0.328 [0.263, 0.392]
	(21)	0.459 (0.028)	
Attitudes toward violence	(22)	0.788 (0.017)	0.723 [0.679, 0.767]
	(23)	0.654 (0.019)	
	(25)	0.666 (0.018)	

*N = 1,810; *p* < 0.001 (one-tailed test) for all factor loadings. Standard errors in parentheses. 95% confidence intervals in brackets. A CFA model without cross-loadings was used to obtain omega reliability coefficients (McDonald, 1999).*

**Table 3:** Correlations between values, attitudes toward interpersonal violence, and interpersonal violent behavior.

	UN	BE	T/C	SE	PO	AC	HE	ST	SD	ATT	VIO
UN	1.0										
BE	0.77***	1.0									
T/C	0.48***	0.33***	1.0								
SE	0.59***	0.63***	0.79***	1.0							
PO	–0.27***	0.03	0.16***	0.22***	1.0						
AC	0.27***	0.50***	0.32***	0.61***	0.82***	1.0					
HE	0.26***	0.67***	0.04	0.46***	0.32***	0.45***	1.0				
ST	–0.10**	0.19***	–0.27***	0.05	0.44***	0.24***	0.53***	1.0			
SD	0.66***	0.92***	0.05	0.59***	0.35***	0.76***	0.82***	0.55***	1.0		
ATT	–0.45***	–0.27***	–0.27***	–0.27***	0.23***	–0.07*	–0.03	0.35***	–0.14**	1.0	
VIO	–0.31***	–0.17**	–0.28***	–0.27***	0.15	–0.01	0.11	0.30***	0.00	0.31***	1.0

*UN, universalism; BE, benevolence; T/C, tradition/conformity; SE, security; PO, power; AC, achievement; HE, hedonism; ST, stimulation; SD, self-direction; ATT, attitudes toward violence; VIO, interpersonal violent behavior; *N* = 1,810; ^∗^*p* < 0.05; ^∗∗^*p* < 0.01; ^∗∗∗^*p* < 0.001 (two-tailed test).*

**FIGURE 3:**
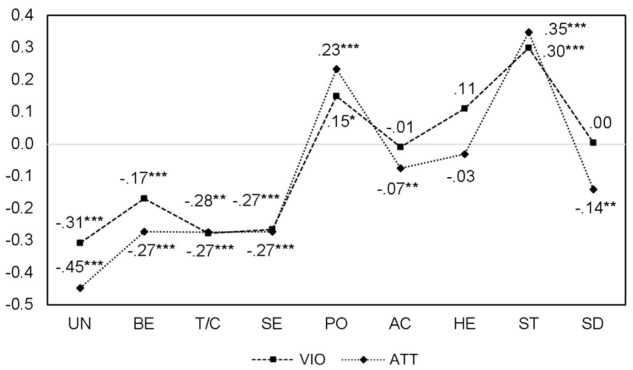
Correlations between values, attitudes toward interpersonal violence, and interpersonal violent behavior. UN, universalism; BE, benevolence; T/C, tradition/conformity; SE, security; PO, power; AC, achievement; HE, hedonism; ST, stimulation; SD, self-direction; ATT, attitudes toward interpersonal violence; VIO, interpersonal violent behavior; *N* = 1,810.

According to the theory, the circular organization of the values can be graphically represented as a sinusoid curve that appears when the associations between values and outside variables are plotted (Schwartz, 1992, p. 54). The correlations between the nine values, attitudes toward violence, and violent behavior are illustrated in **Figure 3**. The curves are interrupted by low correlations with achievement as well as hedonism. Furthermore, the highest (*r* = 0.30/0.35 for stimulation, respectively) and lowest (*r* = -0.31/-0.45 for universalism, respectively) correlations appear for values that are separated by only one value (self-direction) in the circular structure. The partial failure to follow a sinusoid curve indicates that the attitudes toward violence and violent behavior are grounded in multiple motivations. Positive attitudes toward violent behavior and performing violent behavior may be an expression of the pursuit of dominance (power) and of thrill seeking (stimulation). However, these motivations need not occur together. Each alone is a sufficient motivation for regarding violence positively.

### Multivariate Analysis: The Interrelations Between Values, Attitudes Toward Interpersonal Violent Behavior, and Violent Behavior

Next, we examined the impact of values on attitudes toward violence, as well as the impact of attitudes toward violence on violent behavior using structural equation models (Bollen, 1989). We also examined whether, in some cases, values exerted an additional *direct* effect on behavior. We used the software package M*plus* (Muthén and Muthén, 1998–2012) for the analysis. Due to the non-normal character of some items and missing values (Schafer and Graham, 2002), we implemented the full information maximum likelihood estimator (MLR).^16^ We used separate models for each value as a predictor for two reasons. First, using all values simultaneously as predictors may be problematic with the specific data, because the number of predictors would be too high compared to the small number of reported violent events captured by the dependent variable. Focusing on each value separately enables estimates with more statistical power to be produced. Second, focusing on each value is a common procedure in value research (known as the so-called “magnifying glass strategy”; see e.g., Piurko et al., 2011; Cieciuch and Schwartz, 2012; Cieciuch et al., 2014). Thus, we estimated separate structural equation models for each value, that is, nine models in total. The basic structure of the following models is depicted in **Figure 2**. Estimates of the structural parameters including direct and indirect effects are shown in **Table 4**.

**Table 4:** Structural equation modeling results for value-specific models.

		Unstandardized effects		Standardized effects	
Model		direct	indirect	direct	indirect
1	UN → ATT	–0.420 (0.048)***		–0.400 (0.038)***	
	UN → VIO	–0.250 (0.143)*	–0.164 (0.047)***	–0.148 (0.082)*	–0.097 (0.027)***
	ATT → VIO	0.391 (0.110)***		0.242 (0.066)***	
2	BE → ATT	–0.301 (0.075)***		–0.284 (0.043)***	
	BE → VIO	–0.263 (0.152)*	–0.122 (0.040)**	–0.155 (0.089)*	–0.072 (0.019)***
	ATT → VIO	0.405 (0.095)***		0.253 (0.057)***	
3	TRCO → ATT	–0.328 (0.048)***		–0.288 (0.038)***	
	TRCO → VIO	–0.406 (0.167)**	–0.132 (0.037)***	–0.219 (0.083)**	–0.071 (0.018)***
	ATT → VIO	0.402 (0.095)***		0.246 (0.055)***	
4	SE → ATT	–0.291 (0.053)***		–0.329 (0.038)***	
	SE → VIO	–0.373 (0.141)**	–0.106 (0.031)***	–0.257 (0.082)***	–0.073 (0.020)***
	ATT → VIO	0.365 (0.100)***		0.222 (0.060)***	
5	PO → ATT	0.389 (0.067)***		0.216 (0.041)***	
	PO → VIO	0.266 (0.233)	0.172 (0.047)***	0.093 (0.077)	0.060 (0.017)***
	ATT → VIO	0.443 (0.091)***		0.279 (0.052)***	
6	AC → ATT	–0.071 (0.036)*		–0.066 (0.033)*	
	AC → VIO	0.013 (0.122)	–0.033 (0.018)*	0.008 (0.072)	–0.020 (0.010)*
	ATT → VIO	0.473 (0.089)***		0.301 (0.050)***	
7	HE → ATT	0.058 (0.045)		0.048 (0.037)	
	HE → VIO	0.219 (0.166)	0.027 (0.021)	0.113 (0.084)	0.014 (0.011)
	ATT → VIO	0.468 (0.089)***		0.295 (0.050)***	
8	ST → ATT	0.224 (0.054)***		0.173 (0.040)***	
	ST → VIO	0.285 (0.223)	0.099 (0.030)***	0.138 (0.105)	0.048 (0.014)***
	ATT → VIO	0.442 (0.089)***		0.278 (0.052)***	
9	SD → ATT	–0.179 (0.086)*		–0.104 (0.049)*	
	SD → VIO	0.087 (0.260)	–0.086 (0.044)*	0.032 (0.095)	–0.032 (0.016)*
	ATT → VIO	0.478 (0.092)***		0.304 (0.052)***	

*UN = universalism, BE = benevolence, TRCO = tradition/conformity, SE = security, PO = power, AC = achievement, HE = hedonism, ST = stimulation, SD = self-direction, ATT = attitude toward interpersonal violence, VIO = interpersonal violent behavior, all coefficients aimed at VIO are probit coefficients, *N*= 1,810. ^∗^*p* < 0.05; ^∗∗^*p* < 0.01; ^∗∗∗^*p* < 0.001 (one-tailed test). Standard errors in parentheses. Total effects can be obtained as the sum of direct and indirect effects.*

First, **Table 4** clearly demonstrates that – as expected – attitudes toward violence display a positive and significant effect on violent behavior irrespective of the type of value included in the model. Second, and consistent with our propositions, universalism, benevolence, tradition/conformity, and security displayed negative and significant (*p* < 0.01) effects on attitudes toward violence. These values had also negative and significant direct effects on interpersonal violent behavior. The strongest negative effect was for universalism. Thus, self-transcendence and conservation values, contrary to our propositions, had also a *direct* effect on behavior, and not only an indirect one. Furthermore, and as expected, power and stimulation exerted a positive and significant (*p* < 0.01) effect on attitudes toward violence but no direct effect on behavior. Contrary to the expectations, achievement and self-direction exerted negative, albeit small effects on attitudes toward violence (*p* < 0.05) but not directly on behavior. As expected, hedonism did not display any effect on attitudes toward violence or on interpersonal violent behavior.

Universalism (*p* < 0.05), benevolence (*p* < 0.05), tradition/-conformity (*p*< 0.01), security (*p*< 0.01), achievement (*p* < 0.05), and self-direction (*p* < 0.05) also displayed significant negative *indirect* effects on interpersonal violent behavior. Power and stimulation exerted positive and significant (*p* < 0.01) indirect effects on interpersonal violent behavior.^17^

In sum, our expectations with regard to the effects of values on attitudes toward interpersonal violence and interpersonal violent behavior were mostly supported by the data. Universalism, benevolence, tradition/conformity, and security had the strongest negative effects on attitudes toward interpersonal violence and direct as well as indirect negative effects on interpersonal violent behavior. In line with our expectations, these values can be considered as inhibiting violence. Furthermore, small negative effects on attitudes toward interpersonal violence were found for achievement and self-direction values. Although we did not expect to find any effects for achievement and self-direction, the negative impacts are in line with the motivational goals associated with achievement and self-direction. Personal success (reflected in achievement values) is strongly guided by adherence to conventional standards of behavior, which oppose violent behavior. Similarly, autonomy and freedom (reflected in self-direction values) are seemingly not pursued by any means. As expected, considerable positive effects on attitudes toward interpersonal violence were found for power and stimulation values. Their impact on violent behavior was mainly indirect and mediated through attitudes. Thus, these values can be considered as propelling interpersonal violence. Finally, no significant effects were found for hedonism.

## Discussion

The relevance of human values for the study of the motivational sources of interpersonal violent behavior was investigated in different fields of the social sciences. However, various past studies failed to distinguish values from other dimensions such as attitudes, norms, or beliefs, and only very few systematically assessed the effect of values on violent behavior relying on a value theory. Furthermore, in various studies interpersonal violence was often analyzed in composite indexes of general delinquent behavior rather than in its own right. In the current study, we addressed these gaps in the literature by building upon Schwartz’ theory of basic human values, one of the most systematically developed and empirically tested value theories to date. We used it to explain attitudes toward interpersonal violence and interpersonal violent behavior. We analyzed data for young people in the period of emerging adulthood (drawn from a German study in Duisburg, Germany), which assessed violent behavior and different sources of violent behavior. We used structural equation models to estimate the relationships between values, attitudes, and violent behavior simultaneously.

Our results demonstrated that attitudes toward interpersonal violence displayed a significant relationship with interpersonal violent behavior regardless of the specific value that was analyzed. Thus, attitudes appeared to be an important prerequisite for performing such a behavior. Furthermore, we found that several specific values were associated with attitudes toward interpersonal violence. Some were also directly linked with interpersonal violent behavior (and not only indirectly via the attitudes). Universalism, benevolence, tradition/conformity, and security values were negatively related with attitudes toward interpersonal violence and, in addition, directly related with behavior. Thus, attitudes operated as a partial mediator between these values and violent behavior, and despite the dominant association between attitudes and behavior, values themselves had a significant additional contribution to the explanation of behavior, thus inhibiting violent behavior. Schwartz et al. (2017) argued that values have an effect on behavior especially when the action preserves and promotes the goals that these values express. This effect is particularly strong and potentially direct when the individual gives a high priority to these values. Since violence against others strongly contradicts the motivational goals of universalism, benevolence, tradition/conformity, and security values (e.g., concern for the welfare of others, compliance with social expectations and rules), these values are directly and inversely related with the behavior. These findings provided empirical support for our expectations that values from the dimensions self-transcendence and conservation are particularly relevant for and predictive of interpersonal violence.

By way of contrast and in line with our theoretical considerations, power and stimulation were positively related with attitudes toward interpersonal violence but had no direct relation to behavior. Thus, their relation with interpersonal violent behavior was fully mediated by attitudes toward the behavior. Indeed, pursuing dominance and thrill did not provide motivations, which were strong enough to be directly linked with violent behavior, and the relation of the values with behavior was mediated by attitudes toward violence. Thus, not all values from the dimensions self-enhancement and openness to change propel interpersonal violence. Only insofar as a motivation for dominance and thrill seeking is concerned, as is the case for the values power and stimulation, a positive effect of values on interpersonal violence could be empirically supported by the data. This finding underlines the need to test how the distinctive effect of specific values operates in predicting attitudes toward violence and violent behavior (Schwartz et al., 2017). Achievement and self-direction displayed a smaller negative effect on attitudes toward interpersonal behavior; thus, these values also displayed an inhibiting effect on attitudes toward interpersonal violent behavior. Finally, hedonism showed neither direct nor indirect associations with either attitudes or behavior.

This study is not without limitations. Since values were measured only once, it was not possible to assess the causal pattern between values, attitudes, and behavior. It might very well be the case that the more people engage in violence, the more they become inured to its consequences. Hence, violence may also lead to an assessment of violent acts as less serious and to an adaptation of the value priorities and attitudes. One would need panel data with repeated measures for values, attitudes toward interpersonal violence, and violent behavior to assess the direction of causality more closely or an experimental design using, for instance, a factorial survey (see, e.g., Atzmüller and Steiner, 2010; Dülmer, 2015). The possibility to test equivalent models with contemporaneous effects in the opposite direction is tempting, but deemed inadequate to determine causal dominance (Bollen and Pearl, 2013). Instead, our argumentation for the specified direction of effects relies on sound theoretical reasoning, and we have solid reasons to believe that associations between values, attitudes, and behavior are at least indicative of one possible causal chain (Schwartz et al., 2017). Since values are considered much more abstract and stable than attitudes or behavior, we assumed that the causal direction follows from values to attitudes to behavior (or directly from values to behavior), but we do not exclude the possibility that it might also operate in the other direction (e.g., Vecchione et al., 2016). Hence, the assessment of reversed causality and causal dominance is an important aspect for future research on the topic.

As values were measured only at one point in time, we also did not address the developmental process of values, attitudes, and behavior and how their developmental pattern may be linked in this study. This, too, could be an exciting avenue for future research. In addition, interpersonal violent behavior is a rare event and as such limits the analytical possibilities, which would be otherwise much more flexible for normal data (King and Zeng, 2001). Particularly interpersonal violent behavior (but to a certain extent also attitudes toward such behavior) may have suffered from underreporting, since respondents may have hidden their positive attitude toward violence or violent behavior due to social desirability bias (e.g., Meyerl, 2013). After all, violence is clearly a socially undesirable behavior. This in turn may result in an underestimation of the true effects for some values which are desirable like universalism or benevolence, or with an overestimation for other values that are considered rather undesirable, particularly tradition, conformity, and security (Schwartz et al., 2012). However, the designers of the study took precautions to reduce this problem to a minimum as described in the “Data and Measures” section, for example, by guaranteeing anonymity and avoiding the collection of any personal data that allows identifying specific respondents. Future research may try to address this problem by carefully considering the sensitivity of the questions asked and trying to control for it.

Finally, the 21 items to assess values are limited in their capacity to discriminate between 10 distinct values (Davidov, 2008, 2010; Davidov et al., 2008b). Although the scale worked well in this study, the tradition and conformity values had to be treated as a combined value. Longer versions of the questionnaire (e.g., PVQ-40, PVQ-RR) are needed to fully capture the continuum of human values Cieciuch and Schwartz, 2012).

In spite of these limitations, the current study demonstrated that values, attitudes toward violence, and violent behavior are linked, and that values can predict violent behavior, either indirectly via attitudes toward violence or directly. Indeed, although interpersonal violence is (fortunately) a rare event, abstract values – in combination with attitudes toward interpersonal violent behavior – are able to explain interpersonal violent behavior to a large extent and deepen our understanding of its motivational sources.

## Ethics Statement

The study was carried out doing secondary data analysis of a project at the University of Bielefeld. For further details, one may contact the principal investigator Jost Reinecke (jost.reinecke@uni-bielefeld.de). No data were collected by the authors themselves for the preparation of the submitted study.

## Author Contributions

All authors listed have made a substantial, direct and intellectual contribution to the work, and approved it for publication.

## Conflict of Interest Statement

The authors declare that the research was conducted in the absence of any commercial or financial relationships that could be construed as a potential conflict of interest. The reviewer GM and handling Editor declared their shared affiliation and their involvement as co-editors in the Research Topic, and confirm the absence of any other collaboration.

## Footnotes

1 Schwartz et al. (2012) have since refined the theory. The new version of the theory differentiates between more values but still allows using wider value categories as in its original version, because it presents values as a continuum. However, because the empirical part in our study refers to the original version of the theory from 1992, we will not make further reference to the refined version.

2 These characteristics distinguish values from personality traits and attitudes (Roccas et al., 2002; see also Iser and Schmidt, 2005).

3 Furthermore, the influence of values on attitudes and behavior can be seen as a “tradeoff” between each value and opposing values (Schwartz et al., 2017). In other words, attitudes and behavior may be motivated (or demotivated) by and grounded in multiple values.

4 Previous studies which primed values supported such causal influence by showing that value manipulations influence subsequent behavior in value-consistent directions and energizes action (e.g., Verplanken and Holland, 2002; Maio et al., 2009; Sagiv et al., 2011; Maio, 2015; Schwartz et al., 2017). For instance, when achievement was primed, individuals tried to perform better, when self-direction was primed, individuals made stronger efforts to look for information, or when benevolence was primed, individuals tried harder to help others. We will return to this point in our final section.

5 Theoretical foundations of research on aggression exist in various domains of personality and social psychology. We refer readers to the relevant literature on cognitive, social learning, script, and social interaction theories (for an overview see Anderson and Bushman, 2002; DeWall et al., 2011).

6 We would like to note that the relationship between the value security and support of violence may be positive when state authorities use violence to restore social order or to protect national security (Sundberg, 2014). However, such a situation is beyond the scope of our study because our attitudinal measures tap into support of individual interpersonal violent behavior.

7 Principal investigators are Klaus Boers (University of Münster, Germany) and Jost Reinecke (University of Bielefeld, Germany). The study is funded by the German Research Foundation (Deutsche Forschungsgemeinschaft). See www.crimoc.org for further information.

8 The study investigated additional cohorts, partly in different cities. The analysis in this paper refers, however, only to the Duisburg seventh-grade cohort.

9 Other studies like the Cambridge Study in Delinquent Development (Farrington et al., 2013) or the Rochester Youth Development Study (Thornberry et al., 2003) focused on respondents with a higher risk of delinquent involvement (e.g., males, convicts, individuals from urban areas with high crime rates). This strategy results in higher rates of delinquency, but may limit the generalizability of the findings.

10 A self-generated code that consisted of six time-invariant personal characteristics was used to link panel waves and grant anonymity. Some participants were not able to reproduce the code in the surveys across waves, and their data could not be linked across time (see Seddig, 2014, 2016).

11 We modeled values as latent variables using confirmatory factor analysis (CFA, see Brown, 2015). Therefore, we did not use centered value scores but rather the raw scores, as recommended by Schwartz (2009) for value models with latent variables. Latent variable variances reflected interindividual differences in the prioritization of a particular value.

12 Although intimidation does not cause direct physical harm, we decided to keep the item because it involves the threat of violence.

13 Since there were only two cases in the data reporting robbery, our models explain first and foremost assault.

14 Analyzing these data as (Poisson or negative binomial) count variables is not applicable because very few people reported more than one offense. Therefore, we treated the data as dichotomous (non-offenders vs. offenders). However, due to the small number of reports in each wave, we calculated a composite of self-reports for both waves. Participants who reported an offense in both years were only considered once, and the variable contained 49 offenders in total.

15 χ2 = chi-square; df = degrees of freedom; RMSEA = root mean square error of approximation; CFI = comparative fit index. For cut-off criteria, see Hu and Bentler (1999) and Marsh et al. (2004). Additionally, cross loadings and residual covariances were specified to fit the model with all single values to the data (see Davidov et al., 2008b). Detailed outputs for all the models reported in this paper can be obtained from the first author upon request.

16 Alternatively, the WLSMV estimator can be used in Mplus to account for non-normal ordinal data (Flora and Curran, 2004). Analyses with WLSMV revealed no substantial differences.

17 To test the robustness of our results we performed an analysis where we also controlled for gender. All effects remained significant and pointed to the same direction. In general, men reported more violent acts and had more positive attitudes toward interpersonal violence than women. Furthermore, men attributed greater importance to power, stimulation, hedonism, and achievement values than women. Women attributed greater importance to universalism, benevolence, security, and conformity values than men.
